# A Critical Evaluation of the Influence of the Dark Exchange Current on the Performance of Dye-Sensitized Solar Cells

**DOI:** 10.3390/ma9010033

**Published:** 2016-01-08

**Authors:** Rodrigo García-Rodríguez, Julio Villanueva-Cab, Juan A. Anta, Gerko Oskam

**Affiliations:** 1 Departamento de Física Aplicada, Centro de Investigación y de Estudios Avanzados del Instituto Politécnico Nacional (CINVESTAV-IPN), Mérida, Yucatán 97310, Mexico; 2 Instituto de Física, Benemérita Universidad Autónoma de Puebla (BUAP), Puebla, Puebla 72570, Mexico; juliovc@ifuap.buap.mx; 3 Nanostructured Solar Cells Group, Department of Physical, Chemical and Natural Systems, Universidad Pablo de Olavide, Seville ES-41013, Spain; anta@upo.es

**Keywords:** dye-sensitized solar cells, continuity equation, dark current, exchange current density, optimal film thickness, super-position principle

## Abstract

The influence of the thickness of the nanostructured, mesoporous TiO_2_ film on several parameters determining the performance of a dye-sensitized solar cell is investigated both experimentally and theoretically. We pay special attention to the effect of the exchange current density in the dark, and we compare the values obtained by steady state measurements with values extracted from small perturbation techniques. We also evaluate the influence of exchange current density, the solar cell ideality factor, and the effective absorption coefficient of the cell on the optimal film thickness. The results show that the exchange current density in the dark is proportional to the TiO_2_ film thickness, however, the effective absorption coefficient is the parameter that ultimately defines the ideal thickness. We illustrate the importance of the exchange current density in the dark on the determination of the current–voltage characteristics and we show how an important improvement of the cell performance can be achieved by decreasing values of the total series resistance and the exchange current density in the dark.

## 1. Introduction

The development of new sources of energy that can be economically viable and environmentally friendly is one of the most important goals in current scientific research. Solar energy is one of the most promising alternatives as a clean energy source as the Sun provides the Earth in one hour with as much energy as all mankind uses in one year [1,2]. Development of this solar energy includes the development of third generation solar cells. This type of solar cell includes dye-sensitized solar cells (DSCs) [3,4,5,6] and the recently discovered perovskite-based solar cells [7,8,9,10]. Dye-sensitized solar cells are photoelectrochemical systems that represent an interesting option in the development of new technology in the photovoltaics field related to the use of earth abundant materials, potentially low cost, and simplicity of manufacturing techniques. The DSC consists of a transparent conductive oxide (TCO) on which a layer of a nanostructured, mesoporous semiconductor, usually TiO_2_, is deposited. TiO_2_ has a large bandgap (3.2 eV) and it does not absorb visible light, hence, the semiconductor is sensitized with a molecular dye, which can be an organometallic ruthenium complex or a purely organic dye. The solar cell is sealed with a thermoplastic polymer and another TCO acting as counter electrode, which is usually activated with an electrocatalyst consisting of small Pt islands or carbon materials. The solar cell is filled with an electrolyte solution based on an organic low-viscosity solvent and a redox couple, classically I^−^/I_3_^−^ or the newer, higher-performance Co^2+/3+^(bpy)_3_ and related couples [11,12,13,14]. Recent advances in dye-sensitized solar cells include the development of flexible fiber/wire-shaped solar cells [7,15], co-sensitization for increasing light absorption, and performance [11,16] and copper-based redox couples [17,18,19]. This photoelectrochemical system is very complex, combining physical and chemical properties of inorganic nanomaterials, optoelectronic properties of molecular organic dyes, and non-aqueous electrochemistry, and in order to understand the phenomena that take place in the solar cell and to achieve improved performance, the study of interrelated parameters using advanced characterization techniques is essential. 

One of the factors that affect DSC performance is the thickness of the nanostructured, mesoporous, TiO_2_ film [20,21,22,23,24,25,26,27]. The exposed TiO_2_ surface area is proportional to the thickness, hence, a thicker TiO_2_ film allows for more dye to be adsorbed resulting in an increase in light harvesting efficiency; however, the surface is also the main source of recombination sites, which directly affects the charge collection efficiency. Various studies have shown that the efficiency of the DSC increases with thickness of the film until it reaches a critical value, and then decreases, thus defining an optimal film thickness. The optimal thickness is generally reported to be around 10 µm [20,23,26], although 15–20 µm has also been reported as the ideal thickness [21,22,25,27]. It has been proposed theoretically that this dependence on thickness is a consequence of a balance between absorption of light and photogeneration of electrons and dark electron transfer processes in the cell [28]. In describing dark processes, we need to take into account a variety of parameters. One of these parameters is the exchange current density in the dark, *J*_0_, and several groups have analyzed the influence of *J*_0_ on the performance of the solar cell [27,28,29,30,31]. Assuming that *J*_0_ ∝ *d*, where *d* is the semiconductor film thickness, Zhu and coworkers [28] determined theoretically the effect of the film thickness dependence of *J*_0_ on the performance of DSCs. Using this assumption they concluded that, as the semiconductor film thickness increases, the solar cell becomes more efficient due to better absorption of light and a larger photogenerated current, but after reaching a thickness of 10 μm or more, the processes in the dark have more relevance in the performance of the cell and ultimately limit the efficiency of the device; the optimal film thickness was found to be around 10 μm. In spite of this, the relationship between *J*_0_ and *d* has not been yet unambiguously demonstrated and has not been verified experimentally.

In this work, we have determined values of *J*_0_ by means of both steady state current–voltage measurements analyzed using the diode equation and small perturbation techniques, such as electrochemical impedance spectroscopy (EIS), intensity-modulated photovoltage spectroscopy (IMVS) and intensity-modulated photocurrent spectroscopy (IMPS). Using a continuity equation model for the total electron density, we analytically derive a relationship between *J*_0_ and the recombination time constant and electron density in the dark, which are parameters that can be determined experimentally using small perturbation measurements; the relationship was corroborated experimentally. We also describe the influence of other parameters on the determination of the optimal film thickness in DSCs according to the diode equation. 

## 2. Theoretical Background

### 2.1. Determination of J_0_ from Steady State Measurements

The current voltage response of a solar cell can be approximated as the sum of the short circuit photocurrent and the dark current as a function of applied voltage; this method is known as the superposition approximation. Although the reverse current which flows in response to voltage in an illuminated cell is not formally equal to the current which flows in the dark, the approximation is reasonable for many photovoltaic materials. The sign convention for current and voltage in photovoltaics is such that the photocurrent is positive. With this sign convention and for a non-ideal diode, the net current density in the cell is [32,33,34]:

(1) $$J=J_{SC}-J_{0}\left[exp\left(\frac{qV}{mk_{B}T}\right)-1\right]$$

where *J_SC_* is the short-circuit photocurrent density, *J*_0_ is the exchange current density in the dark, *k_B_* is the Boltzmann constant, *T* is temperature, *q* is the elementary charge, and *m* is the ideality factor of the diode equation. The value of *m* depends on the order of the recombination reaction rate in electrons and oxidized species in the electrolyte, and the distribution of recombination sites [35,36]. In addition, the value of *J*_0_ is related to the rate constant of the recombination reaction, the film thickness, the order of reactions of electrons and oxidized redox species, and the background concentrations of electrons in the TiO_2_ and oxidized redox species in the electrolyte [28]. The second part of Equation (1) represents the dark current density. At sufficiently high bias, the two currents cancel, corresponding to open-circuit conditions, while at zero bias the cell produces its maximum current, corresponding to short-circuit conditions. Together with series resistance losses, the balance between transport and recombination determines the shape of the current–voltage curve of the device [33,37].

If only the resistance losses relating to current are considered, the expression for the net current density (*J_net_*) of a DSC can be expressed as:

(2) $$J_{net}\left(V\right)=J_{SC}-J_{0}\left\{exp\left[\frac{q\left(V+J_{net}R_{S}^{total}\right)}{mk_{B}T}\right]-1\right\}$$

where *J_net_* is the net photocurrent density, *R_s_^total^* is the total series resistance of the solar cell and *V* is the applied voltage. From the last equation we can obtain:

(3) $$\frac{dV}{dJ_{net}}=-R_{S}^{total}+\frac{mk_{B}T}{q}\cdot \frac{1}{J_{net}-J_{sc}-J_{0}}$$

The dark exchange current density, *J*_0_, is normally orders of magnitude smaller than the sum of *J_net_* and *J_SC_* and, therefore, can be neglected in this equation [28,38]. Isolating the *J*_0_ term in Equation (2) and under open circuit conditions (*J_net_* = 0), we obtain the following expression:

(4) $$J_{0}=\frac{J_{sc}}{exp\left(\frac{qV_{oc}}{mk_{B}T}\right)-1}$$

From Equation (4) we can obtain a value for *J*_0_. Considering no recombination losses at short-circuit conditions, the value of *J_SC_* will be equal to the light absorbed:

(5) $$J_{SC}=q\phi T_{FTO}\left[1-exp\left(-\alpha_{abs}d\right)\right]$$

where Ф is the incident photon flux, *T_FTO_* is the effective transmittance of the FTO substrate, and α*_abs_* is the effective absorption coefficient of the solar cell. Assuming (i) a collection efficiency of over 99% (η_cc_ ≥ 99%), (ii) *J*_0_ ∝ *d*, (iii) ohmic losses have been corrected for and light scattering, (iv) reflection at the counter electrode, and (v) light absorption by the iodide electrolyte and TiO_2_ are negligible [28], the diode equation can be expressed as:

(6) $$J\left(V\right)=-J_{00}d\left[exp\left(\frac{qV}{mk_{B}T}\right)-1\right]+q\phi T_{FTO}\left[1-exp\left(-\alpha_{abs}d\right)\right]$$

where *J*_00_ is the exchange current density per unit film thickness. The first and second terms on the right side of the last equation denote the contributions to *J(V)* in the dark and in the light, respectively [28]. 

### 2.2. Determination of J_0_ from Small Perturbation Techniques

The electron transport and recombination properties of the DSC have been investigated using a continuity equation for electrons, which can either be expressed in terms of the free electron density in the conduction band, including a separate term for the trapping and detrapping processes, or in terms of the total electron density; it has been shown that these two approaches are mathematically equivalent under “quasi-static conditions” [33]. It is convenient to express the continuity equation in terms of the total electron density in the photoanode, *n*, as this is an experimentally accessible quantity: the total electron density includes both free and trapped electrons and can be determined experimentally by charge extraction measurements. Assuming 1-dimensional geometry for electronic processes in the photoanode, the continuity equation can be written as [33]:

(7) $$\frac{\partial n\left(x,t\right)}{\partial t}=\frac{\partial }{\partial x}\left[D_{n}\left(n\left(x\right)\right)\frac{\partial n\left(x\right)}{\partial x}\right]+G\left(x\right)-k_{n}\left(n\left(x\right)\right)n\left(x,t\right)$$

which is the continuity equation in terms of the total electron density, *n*, taking the approximation that a quasi-static equilibrium holds between free and trapped electrons. Here, *x* is the distance to the working electrode, *G(x)* is the volume generation rate and the trapping-detrapping dynamics are implicitly considered in the form of an effective diffusion coefficient, *D_n_*, and an effective recombination constant, *k_n_*, which are a function of the total electron density. Another advantage of using Equation (7) is that the transient and stationary behaviors of the device are described with a single continuity equation [33], so this equation can also be used in the time-dependent analysis. 

The photovoltage is related to the total density accumulated in the semiconductor as [33]:

(8) $$n=n_{0}exp\left[\frac{\alpha qV}{k_{B}T}\right]$$

where *n*_0_ is the total electron density in the dark and α is a parameter that reflects the average energy of the exponential distribution of trap states below the conduction band. This parameter can be estimated from capacitance data obtained from impedance measurements. In the continuity equation (Equation (7)), the last term represents the recombination term, in which *k_n_* stands as a small-perturbation recombination constant, which can be obtained directly by small perturbation techniques. The recombination constant represents all possible recombination processes, including recombination with the dye, and can be expressed as [33]:

(9) $$k_{n}\left(n\right)=k_{0}{\left(\frac{n}{n_{0}}\right)}^{\frac{\beta -\alpha }{\alpha }}$$

where *k*_0_ corresponds to the recombination constant in the dark and β is the recombination reaction order with respect to free electrons, corresponding to the inverse of the ideality factor, *m*. From Equations (8) and (9), we obtain an expression for the recombination term in the continuity equation:

(10) $$k_{n}n=k_{0}n_{0}exp\left[\frac{\beta qV}{k_{B}T}\right]=U$$

The recombination current density (*J_rec_*) at the open circuit can be approximated as [30,37]:

(11) $$J_{rec}=Uqd=J_{0}exp\left[\frac{\beta qV}{k_{B}T}\right]$$

where *U* is the recombination term of the continuity equation. The recombination current density, *J_rec_*, is a useful parameter to describe the flux of electrons lost from the TiO_2_. It gives the total rate of loss over the full TiO_2_ film thickness. As the cell voltage increases, the recombination current also increases as expected due to the increasing total charge concentration, *n*, in the TiO_2_ [39]. Combining Equations (10) and (11), we obtain an expression for the exchange current density in the dark:

(12) $$J_{0}=k_{0}n_{0}qd$$

The values of *k*_0_ and *n*_0_ can be obtained from small perturbation techniques (Figures S1 and S2, Supplementary Materials), which allows us to determine the value of *J*_0_ and to compare its value with the value obtained from steady state measurements, in particular, the current–voltage curve.

## 3. Experimental Section

### Elaboration and Characterization of Dye-Sensitized Solar Cells

The procedure to prepare a TiO_2_ screen printing paste was a variation of a standard procedure. An amount of 0.2 mol of acetic acid was added all at once to 0.2 mol of titanium iso-propoxide and vigorously stirred for 15 min at room temperature. The mixture of titanium iso-propoxide and acetic acid was added drop-wise into a flask with 290 mL water with vigorous stirring. The final mixture was stirred at 700 rpm for 40 min. After adding 4 mL of nitric acid, the mixture was heated from room temperature to 80 °C within 40 min and subsequently peptized for 75 min. Water was then added to the cooling liquid mixture to adjust the volume to 370 mL. Aliquots from the resultant mixture were hydrothermally treated at 200 °C for 12 h in a Teflon-lined titanium autoclave (Parr Instruments, Moline, IL, USA). After the heating process, the mixture was sonicated for 1 h, centrifuged, and washed with ethanol. The final material was a colloid with 40 wt% TiO_2_. An amount of 1.8 g of the colloid was dissolved in 9 ml ethanol and mixed with 7.3 g terpineol; the resulting mixture was sonicated for 1 h. This mixture was added drop-wise to a solution of 0.9 g ethyl cellulose in 10 mL ethanol. The final mixture was sonicated for 1 h and the excess ethanol was removed with the aid of a rotary evaporator [20].

To prepare the DSC working electrodes, fluorine-doped tin oxide (FTO) glass is used as current collector (Pilkington TEC 15, Xop Glass, Castellón, Spain). The FTO glass was cleaned in a detergent solution (Alconox) using an ultrasonic bath for 10 min, and then rinsed with deionized water. The FTO glass was then submerged sequentially in deionized water, ethanol, and iso-propanol and treated in an ultrasonic bath for 10 min. The FTO substrate glasses were heated up to 450 °C for 30 min in order to eliminate all the organic residues. A layer of TiO_2_ paste was coated on the FTO by screen printing and then dried for 10 min at 120 °C; five layers of TiO_2_ were deposited using this method, after which the films were heated following the temperature ramp described in Figure S3 in the Supplementary Materials. After the heat treatment, another five layers were deposited onto the previously deposited films in order to increase the thickness of the film. After deposition, the films underwent the previously mentioned heat treatment. This procedure was repeated until the film reaches the desired thickness. Films of 5, 10, 15, 20, 25, and 30 layers were elaborated. The application of the temperature ramp is necessary every five depositions in order to prevent failures and cracks in the semiconductor film. All the electrodes were, once again, heated according to the temperature ramp described in Supplementary Materials, but they were taken to a final constant temperature of 80 °C. The electrodes at 80 °C were immersed in a solution of 0.5 mM N-719 dye in ethanol and kept at room temperature for 24 h in the dark to assure complete sensitizer uptake. To prepare the counter electrodes, two holes were drilled in FTO-covered glass electrodes (Pilkington TEC 8, Xop Glass). The perforated counter electrodes were cleaned following the same procedure as used for the working electrodes. The counter electrode was covered with Pt electrocatalyst by spreading a drop of Platisol (Solaronix, Aubonne, Switzerland) and sintering at 400 °C for 5 min. The sensitized TiO_2_ electrode and Pt-catalyzed counter electrode were assembled into a sandwich-type cell and sealed with Surlyn polymer of 60 µm thickness by applying mechanical pressure and a temperature of 215 °C for 15 min. The electrolyte solution was introduced trough the two small holes in the back of the counter electrode. The electrolyte composition was 0.6 M DMII (1,2-dimethyl-3-propylimidazolium iodide), 0.1 M LiI, 0.1 M GuSCN (guanidinium thiocyanate), 0.05 M I_2_, and 0.5 M TBP (tert-butyl pyridine) in a mixture of acetonitrile and valeronitrile (volume ratio 85:15). In order to have good electrical contact for the connections to the measurement set-up, the edges of the FTO outside the cell were covered with a conductive silver paint (SPI-High Purity Silver Paint) [20]. A picture of the DSCs with a different number of coatings is presented in Figure S4 in Supplementary Materials.

The thickness of the TiO_2_ nanoparticulate film was measured using a KLA Tencor AlphaStep D-120 profilometer (KLA Tencor, Milpitas, CA, USA). The crystal structure and average particle size of the TiO_2_ films were determined by X-ray diffraction (XRD) using a Siemens D-5000 (Siemens, Munich, Germany) with CuKα radiation. Photovoltaic characterization was performed using a set-up consisting of a 450 W ozone-free Xe-lamp (Oriel, Newport Corporation, Santa Clara, CA, USA) with a water filter, calibrated to an irradiance of 100 mW cm^−2^ on the surface of the solar cell using an Air Mass 1.5 Global (AM 1.5G) optical filter (Newport Corporation); the intensity was calibrated using a certified 4 cm^2^ monocrystalline silicon reference cell with incorporated KG-5 filter. Photovoltaic characterization with blue, green, and red light LED illumination (467, 525, and 625 nm wavelength, respectively) was also employed. The scan rate for all the current–voltage curves was 0.01 V/s. Current–voltage curves (I–V), electrochemical impedance spectroscopy (EIS), intensity modulated photocurrent spectroscopy (IMPS) and intensity modulated photovoltage spectroscopy (IMVS) were recorded with an Autolab PGSTAT302N/FRA2 set-up (Metrohm Autolab, Utrecht, The Netherlands). The evolution of the electron transport process in the cell was investigated using EIS, both in the dark and under red light LED illumination (625 nm). An AC amplitude of 10 mV was applied and the frequency range was from 0.1 Hz to 100 kHz. The electrochemical impedance spectrum was analyzed using Z-View software with the aid of the equivalent circuit proposed by Fabregat-Santiago *et al*, and is presented in Figure S5 in the Supplementary Materials [20,31]. IMPS and IMVS measurements were performed at modulation frequencies between 1 mHz and 10 kHz. A red LED (625 nm) was used to illuminate the sample from the substrate side and it served both as the bias illumination and the small sinusoidally modulated probe beam. 

## 4. Results and Discussion

**Figure 1:**
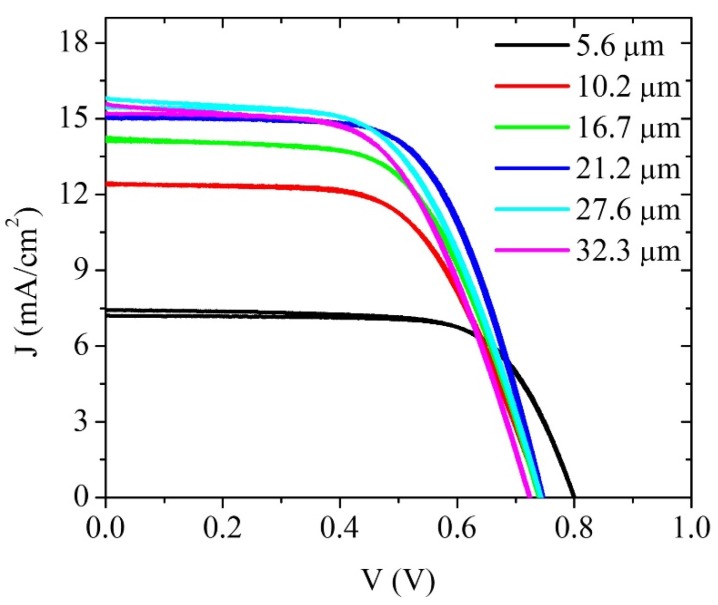
Current–voltage curves for a selection of DSCs as a function of TiO_2_ film thickness.

A series of solar cells was fabricated using screen printed TiO_2_ films of different thicknesses, and the films and the solar cells were characterized using a variety of techniques. The corresponding experimental results of profilometry, Scanning Electron Microscopy (SEM), and X-ray diffraction measurements are shown in the Supplementary Materials in Figures S6–S8. Crack free films were obtained up to a thickness of 32 µm and the TiO_2_ film was crystalline and consisted of pure anatase with an average nanoparticle size of approximately 15 nm (diameter). Figure 1 shows representative J–V curves for dye-sensitized solar cells, illustrating that the current at short circuit tends to increase with film thickness while the open circuit potential decreases. The characteristics of the film and solar cell are summarized in Table S1 in the Supplementary Materials.

**Figure 2:**
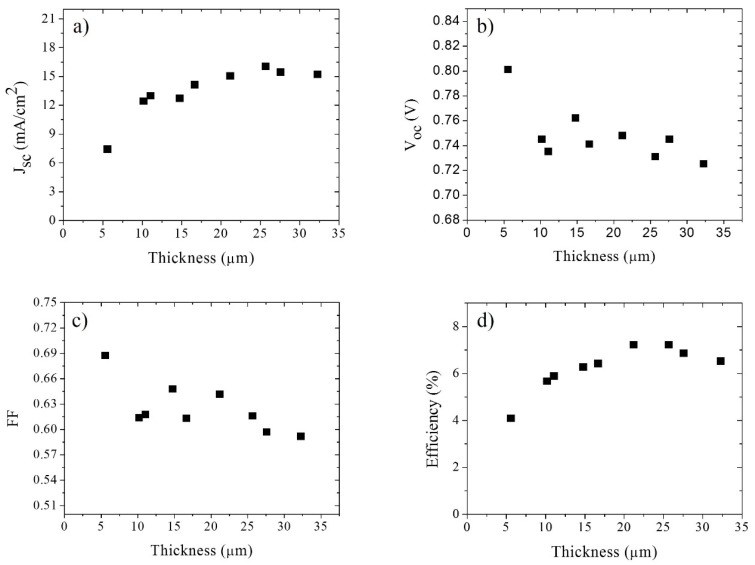
Main parameters of the solar cells as a function of TiO_2_ film thickness; (**a**) *J_SC_*, (**b**) *V_oc_*, (**c**) Fill factor, and (**d**) Overall efficiency.

Figure 2 shows the dependencies of the short circuit current density, *J_SC_*, open circuit photovoltage, *V_oc_*, fill factor, *FF*, and cell efficiency as a function of the film thickness. Upon increasing the film thickness, the exposed TiO_2_ surface area increases, which results in the adsorption of more dye on the film [21,27]. In turn, this leads to an increase of the photogenerated electron flux, which is reflected in the increase of *J_SC_*. Another important feature is that the photocurrent increases with thickness until it reaches a saturation value. This is related to the very limited increase of light absorption with increasing thickness for thick films as most light is already absorbed [24]. We can also observe that as the thickness increases, *V_oc_* decreases. The *V_oc_* is a parameter strongly related with recombination in the cell [20,21,26]. The main loss of current is due to a recombination process between photogenerated electrons in the TiO_2_ and the oxidized redox species in the electrolytic solution [40]. As the surface area of the semiconductor film and contact area with the electrolyte solution increases, the number of recombination pathways increases thus facilitating recombination, resulting in a lower *V_oc_* [21,26,27]. It is important, however, to take other factors into consideration such as dye regeneration, especially for solar cells with large film thickness; although frequently omitted, electron transfer back to the oxidized dye could become an important recombination processes that should not be ignored [39]. The fill factor also diminishes with the increase of thickness. This could be due to inefficient electron injection and/or dye regeneration, especially at high voltages, where regeneration of the dye becomes voltage dependent [41], but also to increasing series resistance losses. At high voltages, the electron concentration in the TiO_2_ can be several times larger than that at short circuit, and this could also increase the probability of undesirable electron transfer to the oxidized sensitizer (S^+^) [42]. As the electron Fermi level is raised, the probability of back reaction between electrons and dye cations increases, resulting in, or as a result of, poor dye-regeneration by the electron donor in the solution [33,39]. In addition, it has been argued that the regeneration of the dye under illumination leads to a higher I_3_^−^ concentration, which should increase the rate of recombination in DSCs [28], although it has been pointed out that poor dye regeneration could ultimately limit the DSCs performances [21]. As mentioned, an increase in series resistance will also decrease the value of fill factor, but, as shown in Figure 4, this parameter varies only slightly with the film thickness in the solar cells. The solar cell efficiency increases with thickness due to a better absorption of light and an increase in photocurrent, until it reaches a maximum value after which it does not change significantly. If the thickness is further increased, recombination will become more important resulting in a decreased solar energy conversion efficiency [40].

**Figure 3:**
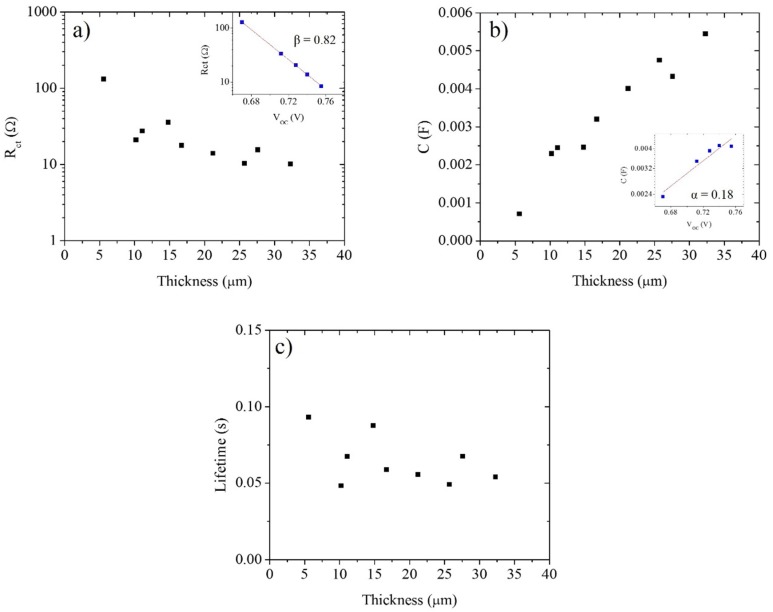
(**a**) Charge transfer resistance, (**b**) chemical capacitance and (**c**) electron lifetime obtained from EIS as a function of TiO_2_ film thickness. The comparison was made at 0.74 V. The insets in (**a**) and (**b**) show the open circuit photovoltage dependence of the charge transfer resistance and chemical capacitance for the 21.2 µm solar cell, respectively; the corresponding values of the parameters α and β are shown.

Figure 3 shows the charge transfer resistance, chemical capacitance and electronic lifetime values obtained by EIS as a function of the TiO_2_ film thickness. The comparison is made at 0.74 V, although the tendency is the same at other voltages. The insets show the dependence of *R_ct_* and *C_µ_* on the open circuit potential under illumination (at different light intensities) for the 21.2 µm cell. The values of the trap distribution parameter α (Equations (8) and (9)) and the recombination reaction order, β, (Equation (9)) are also displayed. Nyquist plots for this cell and the corresponding fitting are illustrated in Figure S9 in Supplementary Materials.

The results show a decrease in *R_ct_* value with increasing thickness, which means that the recombination processes between electrons in the TiO_2_ film and the oxidized species in the electrolyte or dye become more significant as the thickness increases. This result is consistent with the *V_oc_* thickness dependence observed in Figure 2. Since the surface area is proportional to the thickness (if the morphology is independent of the number of layers deposited), *R_ct_* is expected to be proportional to 1/*d*; Figure 2a illustrates that this is the case. The chemical capacitance increases linearly with film thickness and is proportional to the increase in surface area of the mesoporous layer. Although the chemical capacitance increases with thickness, the value of α for the cell remained constant (α ≈ 0.18), indicating that the energetic distribution of trap states stays the same. The electron lifetime is a function of the charge transfer resistance and the chemical capacitance [31,35,43]. Since both have opposite behaviors with respect to increasing electrode thickness, the electron lifetime is essentially independent of electrode thickness [44], as illustrated in Figure 3c. 

**Figure 4:**
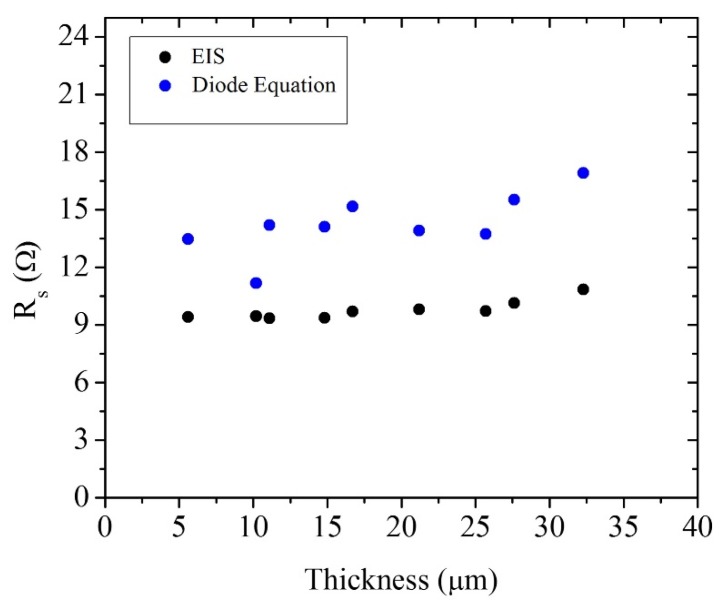
Comparison of the series resistances of the solar cells as a function of TiO_2_ film thickness obtained by two diferent methods: steady state (blue dots) and small perturbation (dark dots) techniques.

In Figure 4, the series resistances of the cells is shown versus film thickness. The values of *R_s_* were determined both by means of the diode equation and by EIS. From Figure 4 it can be concluded that as the thickness increases, the series resistance of the cell increases slightly as well. An increase of the series resistance is expected for thicker cells, hence reducing the efficiency somewhat [21,23,33]. Both experimental methods illustrate that the series resistance does not increase substantially, hence it is not a crucial parameter to determine an ideal film thickness for optimal cell performance. A small but significant difference is observed between the series resistance obtained by the diode equation and by EIS measurements. The series resistance obtained by means of Equation (3) includes the contributions from the active layer corresponding to *r_tr_* and the contribution from the elements connected to the active layer, such as the FTO of the working and counter electrodes, contacts, and wires, and the electrolyte solution; the series resistance obtained by EIS is related only with the series resistance of the FTO [31]. The difference between both values can be observed in Figure 4.

**Figure 5:**
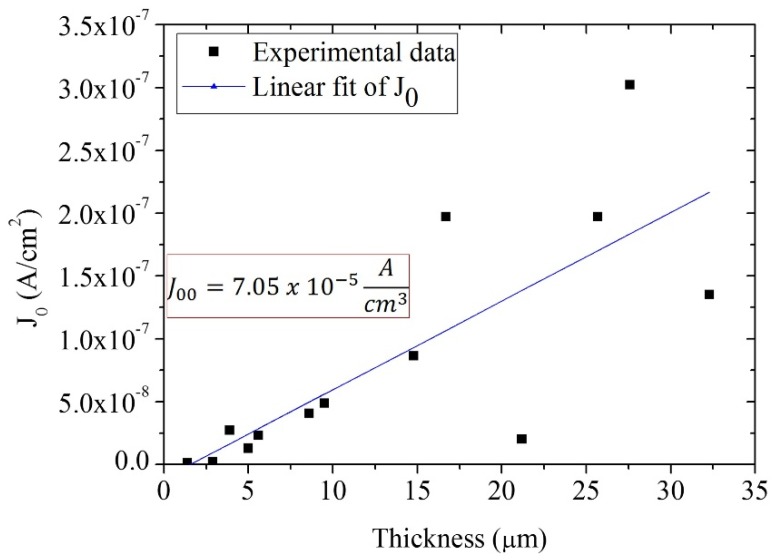
*J*_0_ as a function of film thickness obtained by steady state measurements and the diode Equation (6) under 1 sun illumination. The slope represents *J*_00_.

As the thickness of the semiconductor film increases, the electrochemical system that is the cell becomes more complex and different parameters can have important influence on its performance. As mentioned in the introduction, one of those important parameters is the exchange current density in the dark, *J*_0_, which is directly related with recombination as shown in Equation (11). Using Equation (4), the values of *J*_0_ were determined for all the cells as a function of TiO_2_ film thickness. The experiments were performed under 1 sun illumination and the results are presented in Figure 5. 

Figure 5 shows that the exchange current density in the dark, *J*_0_, is approximately linear with respect to thickness, in agreement with what would be expected from Equation (12). However, it has to be taken into account that the step-wise process that has been used to prepare the films (alternating layer deposition and calcination) can lead to a non-negligible variation of the porosity of the film [45], which could explain the increased scattering at larger thickness. Note, however, that the dependence of *R_ct_*, *C*, and the lifetime on thickness imply that the morphology is essentially independent of thickness.

According to Equation (12), the value of *J*_0_ depends on the rate constant, *k*_0_, for electron transfer from the TiO_2_ to oxidized redox species in solution (recombination) or the oxidized dye, the intrinsic electron density in the film, *n*_0_, and the electrode thickness, *d*; the value of *J*_0_ should become larger with increasing values of these parameters [28]. In a previous study of the influence of thickness in the distributed parameters of the cell by EIS, the results of Jennings *et al.* [44] imply that the electron lifetime and, as a consequence, τ_0_ are independent of electrode thickness. From Equation (S1) in the Supplementary Materials [30,39,40], taking a constant value of porosity and assuming that *Jsc* ∝ *d*, the value of *n*_0_ will be independent of the electrode thickness. Since the value of *k*_0_ and *n*_0_ are independent of electrode thickness, then their product will also be independent of *d*. According to Equation (12), *J*_0_ should follow a linear behavior with film thickness, as was also established by Zhu and coworkers [28]. The results in Figure 5 are in agreement with Zhu’s approach, indicating that from the slope of Figure 5 we can obtain the value of *J*_00_; this value is independent of TiO_2_ film thickness. 

**Figure 6:**
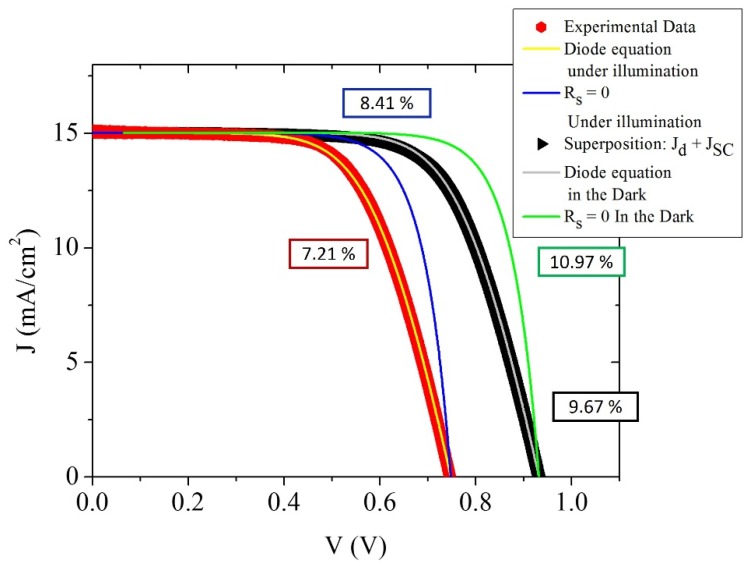
Comparison of the experimental J–V curve (Red dots) under 1 sun illumination obtained for the cell with thickness of 21.2 μm with calculated curves. The dark dots represent the J–V curve for this cell based on the super-position approximation, where the experimental J–V curve measured in the dark in is shifted by the *J_SC_* value obtained under illumination.

In order to determine the influence of *J*_0_ on the performance of a DSC and using the diode equation (Equation (2)), J–V curves for different *hypothetical* cells are presented in Figure 6 and compared to experimental results.

In Figure 6, experimental data obtained for the cell with thickness of 21.2 μm are compared with theoretical J–V curves. The red dots represent the experimental J–V curve for this cell under 1 sun illumination. The dark dots represent the J–V curve for this cell based on the super-position approximation, where the experimental J–V curve measured in the dark in is shifted by the *J_SC_* value obtained under illumination. Using Equation (4), we obtained the values for *J*_0_ for these two curves, assuming the same values for the series resistance and β. Using these parameters and Equation (2), we simulated two J–V curves (yellow and gray lines in Figure 6). Although the conditions in the dark and under illumination are different and this could lead to different values of *R_s_* and β, we kept them constant to evaluate the influence of *J*_0_ in the performance of the cell. We also simulated these two curves with the same values of *J*_0_ and β but with a series resistances equal to zero. For these 4 simulations we calculated the theoretical efficiency of the cells, which is presented in Table 1. 

**Table 1:** Comparison of experimental and simulated J–V curves with different values of *J*_0_ and *R_s_*.

21.2 μm Solar Cell Data	Cell under Illumination	Cell under Illumination; *R_s_* = 0 Ω	Superposition Approximation	Superposition Approximation; *R_s_* = 0 Ω
*J*_0_ (A/cm^2^)	2.01 × 10^−8^	2.01 × 10^−8^	7.2 × 10^−10^	7.2 × 10^−10^
*J*_00_ (A/cm^3^)	9.47 × 10^−6^	9.47 × 10^−6^	3.4 × 10^−7^	3.4 × 10^−7^
*R_s_* (Ω)	13.90	0	13.90	0
β	0.47	0.47	0.47	0.47
Efficiency (%)	7.21	8.41	9.67	10.97

Table 1 shows that the value of *J*_0_ for the cell under dark conditions is lower than under illumination, assuming the same values for *R_s_* and β in both cases. This difference in *J*_0_ reflects the difference in recombination processes under light and dark conditions. The main difference between the conditions in the dark and under illumination is that under illumination, a variety of processes occur involving the dye and the redox couple, thus possibly changing the recombination dynamics. Upon illumination the dye is brought into an excited state, and subsequent ultrafast electron injection results in the generation of oxidized dye molecules. These oxidized dye molecules can be reduced back to their original state by either accepting an electron from the TiO_2_ via the back reaction, which is a recombination process [46], or by accepting an electron from the electron donor in the electrolyte solution, creating an increased local concentration of the electron acceptor. This, in turn, may also affect the recombination kinetics via the electron transfer from the TiO_2_ to the electron acceptor in the solution [47]. The results in Table 1 suggest that these processes could be significant, which is reflected in an increase of the *J*_0_ value under illumination. In the total density approximation (Equation (7)), recombination with the dye is implicitly considered when calculating the recombination constant. In contrast, in the diode equation, this effect is not explicitly considered but leads to a difference in *J*_0_ between dark and illumination conditions. Figure 6 illustrates that this difference in *J*_0_ values leads to a loss of photovoltage of almost 200 mV, which translates to a significant difference in the efficiency of the cell on the order of 2%–3%. This result strongly suggests that dye regeneration is a very important process in the determination of the final performance of the cell, as has been argued by Jennings *et al.* [42]. In addition, the results clearly lead to the conclusion that the superposition approximation does not hold. It is in fact well-known that the light J–V characteristics of DSCs cannot usually be predicted from the sum of the dark J–V and the short-circuit photocurrent (*J_sc_*) [39,42]. Barnes *et al.* examined differences between dark and light J–V curves of DSCs; they found that non-negligible recombination with the oxidized dye, together with local changes in electrolyte concentrations due to current flow, could explain their data [39]. If we take into consideration the influence of the series resistance of the solar cell we can observe in the J–V curves that decreasing *R_s_* results in an increase of the fill factor and the efficiency of the cell. Even for the idealized curve under dark conditions, we can achieve a significant improvement on the efficiency of over 1%. This implies that if we can achieve a very small value of *R_s_* together with a decrease in the value of *J*_0_, the performance of the solar cell could be improved significantly. 

In order to compare the different methods proposed to obtain the exchange current density in the dark, the values of *J*_0_ were also obtained from small perturbation techniques in the dark (EIS) and under illumination (EIS, IMVS, and IMPS), in accordance with Equation (12). The results are presented in Table 2. We also present the values of *J*_0_ obtained from Equation (4) for a solar cell measured under illumination and in the dark, applying the superposition approximation. The most efficient cell (21.2 µm TiO_2_ thickness) was employed for this analysis. From Table 2 we can conclude that the values of *J*_0_ obtained under illumination conditions via the small perturbation methods IMVS-IMPS and the steady-state J–V measurements according to Equation (4) are very similar. We can also observe that the values for *J*_0_ obtained under dark conditions via Equation (4) and the superposition approximation or EIS in the dark lead to practically the same value of *J*_0_, however, *J*_0_ is a factor 300 lower. These results imply that the approximations made with the continuity equation as a function of the total electron density is equivalent to the diode equation with respect to the determination of the *J*_0_ value, and either of the two methods may be employed. Small perturbation methods therefore adequately show that the value of *J*_0_ increases significantly under illumination, since the recombination processes are more significant under these conditions as discussed in the previous paragraph. Note that the results obtained by impedance measurements under illumination led to a different value of *J*_0_ of about one order of magnitude lower, implying that the analysis method may not be accurate or that there are differences in conditions.

**Table 2:** Comparison between the values obtained for *J*_0_ by different methods, both in the dark and under illumination.

21.2 μm Solar Cell Data	Diode Equation under Illumination	Diode Equation with Superposition Approximation	IMVS and IMPS	EIS under Illumination	EIS in the Dark
*J*_0_ (A/cm^2^)	2.01 × 10^−8^	6.75 × 10^−11^	1.17 × 10^−8^	2.44 × 10^−9^	9.44 × 10^−11^
*J*_00_ (A/cm^3^)	9.47 × 10^−6^	3.19 × 10^−8^	5.51 × 10^−6^	1.15 × 10^−6^	4.45 × 10^−8^
*R_s_* (Ω)	13.90	15.45	–	9.80	9.75
β	0.47	0.53	0.79	0.82	0.55

**Figure 7:**
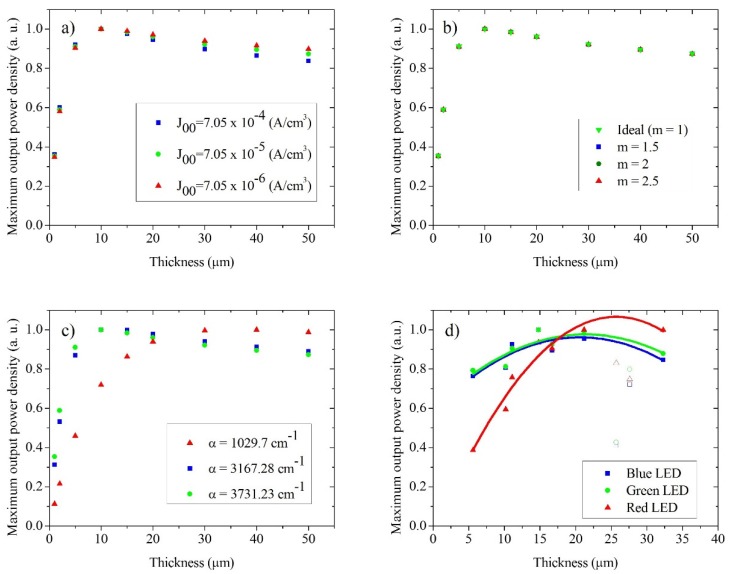
Maximum power density versus film thickness as a function of: (**a**) the value of *J*_00_ (α = 3731.23 cm^−1^; *m* = 2; Φ = 2.62 × 10^17^ cm^−2^·s^−1^; *T* = 0.9; *R_s_* = 0 Ω); (**b**) the value of *m* (α = 3731.23 cm^−1^; *J*_00_ = 7.05 × 10^−5^; Φ = 2.62 × 10^17^ cm^−2^·s^−1^; *T* = 0.9; *R_s_* = 0 Ω); (**c**) the value of α*_abs_* (*m* = 2; *J*_00_ = 7.05 × 10^−5^; *T* = 0.9; *R_s_* = 0; Φ*_C1_* = 2.35 × 10^17^ cm^−2^·s^−1^, Φ*_C2_* = 2.62 × 10^17^ cm^−2^·s^−1^, Φ*_C3_* = 3.14 × 10^17^ cm^−2^·s^−1^); (**d**) Wavelength of light; experimental data obtained for the cells under blue, green, and red LED illumination. Tendency lines are presented and the open dots were not taken into account in the calculation of the fit. Note that the value of α was taken according to the absorption coefficient of the Z907 dye adsorbed onto films at 525 nm (green).

It has been suggested that *J*_0_ ultimately determines the optimal film thickness for the best solar cell performance, because *J*_0_ is an approximately linear function of film thickness [28]. In order to determine the parameters that can influence the optimum film thickness, and using Equation (6) and the experimental values obtained and presented in Figure 5, we have calculated simulated power versus voltage plots as a function of film thickness with different values of *J*_00_, α*_abs_*, *m*, φ, and *T* (Table S2 and Figure S10, Supplementary Materials). From the power density *vs.* voltage plots, the maximum power point was obtained as a function of these parameters, and the results are shown in Figure 7a–d.

Figure 7a shows the maximum power output of solar cells for three values of *J*_00_. It can be observed that the optimum film thickness is around 10 µm, regardless of the value of *J*_00_, implying that, although *J*_00_ is an important parameter for the performance of the DSCs, its value does not determine the optimum film thickness. From Figure 7b, it can be observed that regardless of the value of the ideality coefficient *m*, the behavior of the cells is the analogous to that of Figure 7a. Once again, the optimum film thickness does not change if the value of *m* is modified, and the optimal thickness remains at values of about 10 µm. When analyzing Figure 7b, it is very important to take into consideration that changing the value of β (or *m*) will lead to a change of the *J*_0_ value, as can be observed in Equation 4 in agreement with the results reported by Fabregat-Santiago *et al.* [31]. They found that a decrease in β value would lead to an increase on *J*_0_ and a decrease in the performance and the fill factor of DSCs. In Figure 7c, cells with different values of α*_abs_* are presented. The values of α*_abs_* were taken from the absorption coefficient of the Z907 dye adsorbed to the TiO_2_ film for blue, green, and red light (wavelengths of 467, 525, and 625 nm, respectively). These results were compared with experimental data obtained from J–V curves for the cells under blue, green, and red LED illumination (Figure 7d). It can be observed that the theoretical (Figure 7c) and experimental (Figure 7d) data show a different optimal film thickness for different illumination wavelengths. In spite of the differences in the actual value of optimal film thickness between theoretical and experimental data, the same tendency is observed: changing the value of α*_abs_* changes the ideal thickness of the semiconductor film of TiO_2_. The absorption length, which is the distance for which the light intensity decreases by a factor of 1/*e*, is inversely proportional to the value of α*_abs_*. The value of α*_abs_* for the green and blue wavelengths for the Z907 dye is larger than that for red wavelengths. Therefore, the absorption length for blue and green light is smaller, leading to an optimum film thickness of around 10–15 µm, while for a value of α*_abs_* for red light, the optimum film thickness is around 20–30 µm, approximately 2–3 times larger. This implies that the absorption coefficient of the cell and the irradiation wavelength that is applied to the solar cell ultimately determines the ideal thickness for DSCs. Gonzalez-Vázquez *et al.* established via random walk numerical simulation (RWNS) that the collection efficiency depends on the characteristic optical absorption length in the cells [48]. This result could have implications for the measurements of DSCs, especially using small perturbation techniques, which are often performed with a red LED light illumination. Since white light illumination (operating conditions) corresponds to smaller shorter absorption lengths than red light, the actual diffusion length of the cell under operating conditions could be overestimated when experimentally determined using red LED light illumination.

## 5. Conclusions

The exchange current density in the dark reflects the performance of DSCs, and decreasing its value led to an increase of 200 mV in theoretical cells. Although its value increases linearly with electrode thickness, is very susceptible to changes in porosity, increasing the difficulty to determine it for thick films (*d* ≥ 15 µm). Differences in *J*_0_ values between illumination and dark conditions were obtained, showing the fundamental differences in recombination processes under illumination and dark conditions. This result implies that the superposition approximation will not properly describe the J–V characteristics of the DSCs. Starting with the continuity equation as a function of total electron density, we obtained an expression of *J*_0_ equivalent to the one deduced from the diode equation, and that allows us to determine *J*_0_ via small perturbation techniques. Although *J*_0_ depends on film thickness, it was shown that it does not determine the optimal film thickness of the DSCs, the value of α*_abs_* being the one that determines the optimal film thickness in DSCs.

## Supplementary Materials

The following are available online at www.mdpi.com/1996-1944/9/1/33/s1.
