# Supplementary material for: A Critical Evaluation of the Influence of the Dark Exchange Current on the Performance of Dye-Sensitized Solar Cells
Source: Materials (Basel). 2016 Jan 8;9(1):33. doi: 10.3390/ma9010033 (PMC5456520; doi:10.3390/ma9010033)
Supplement: Supplementary file 1 [file materials-09-00033-s001.pdf]

Supplementary Materials: A Critical Evaluation of the Influence of the Dark Exchange Current on the Performance of Dye-Sensitized Solar Cells

Rodrigo García-Rodríguez, Julio Villanueva-Cab, Juan A. Anta and Gerko Oskam

Figure S1 shows representative IMVS and IMPS results for the solar cells fabricated in this work. In the IMPS curves, the frequency at the apex of the semi-circles decreases with decreasing voltage; this frequency corresponds to the inverse of the electron lifetime, hence, the lifetime increases with decreasing voltage as expected. In the IMPS curves, the same trend is observed. The time constant associated with IMPS measurements is the transport time, which is inversely proportional to diffusion coefficient. This indicates that the electron diffusion coefficient decreases with decreasing voltage, as has been reported previously [1].

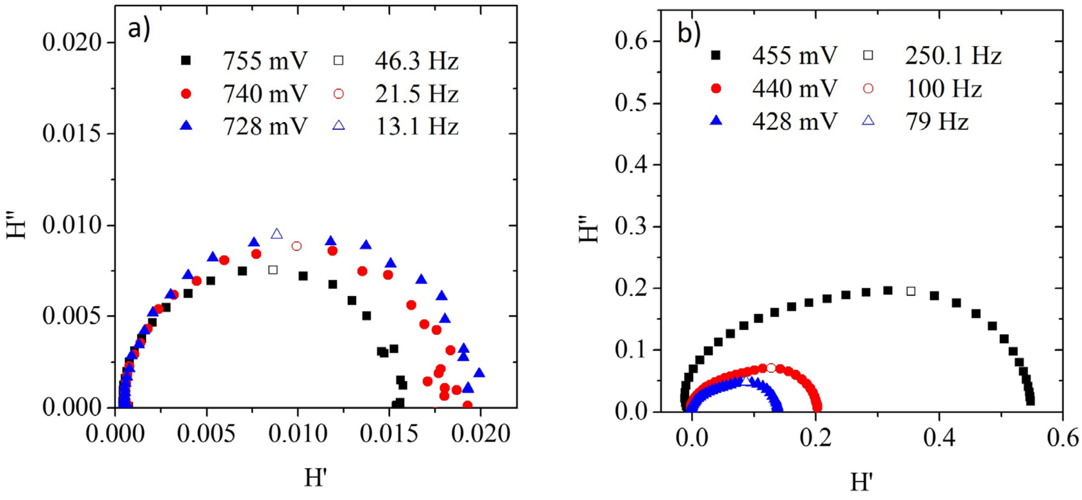

Figure S1. (**a**) IMVS and (**b**) IMPS plots for the 21.2 μm solar cell. The values of the frequency at maximum transfer function point are displayed. The voltages presented in the IMVS curves correspond to the open circuit photovoltage, while in the IMPS curves the voltages correspond to the position of the quasi-Fermi level in the TiO_2 film under short-circuit conditions; the voltages were modulated by changing the light intensity.

k_0 and n_0 Determination by IMVS and IMPS Techniques

IMVS measures the modulation of the photovoltage at open circuit in response to the modulation of the incident light intensity. For pseudo first-order kinetics, the decay of the open-circuit photovoltage is determined by a single time constant which corresponds to the lifetime, τ_r, for recombination of electrons with oxidized species, either the oxidized dye or the electron acceptor in the electrolyte [2]. The recombination constant, k, can be obtained as the inverse of the electron lifetime measured by EIS or IMVS [1,3,4]. From a semi logarithmic plot between electron lifetime and the photovoltage (Figure S2), we can obtain the value of τ_0 and, consequently, of k_0. The mean electron concentration (n) within the TiO_2 can be found according to [5–7]:

$$n = \frac{J_{sc}\tau_r}{qd(1-\theta)} \tag{S1}$$

where J_{sc} is the short circuit current density at the measurement conditions, q is the elementary charge, d is the thickness of the cell and θ is the porosity of the TiO_2 film. For our calculations, we assume a θ value of 0.6. From Equation (8) we have a relationship between the mean electron concentration within the TiO_2 and the photovoltage. Using a semi logarithmic plot between Ln n against V we obtain a straight line and from the intercept with the y-axis we can obtain the value of n_0 (Figure S2).

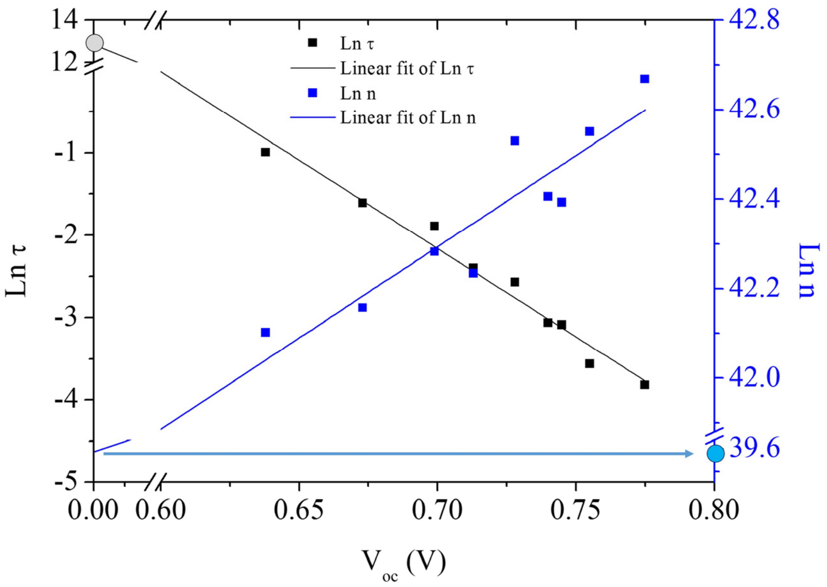

Figure S2. Determination of k_0 ($1/\tau_0$, black dots) and n_0 (blue dots) by extrapolation. The intercept at V_{oc} = 0 V is shown by a circle on the respective axis.

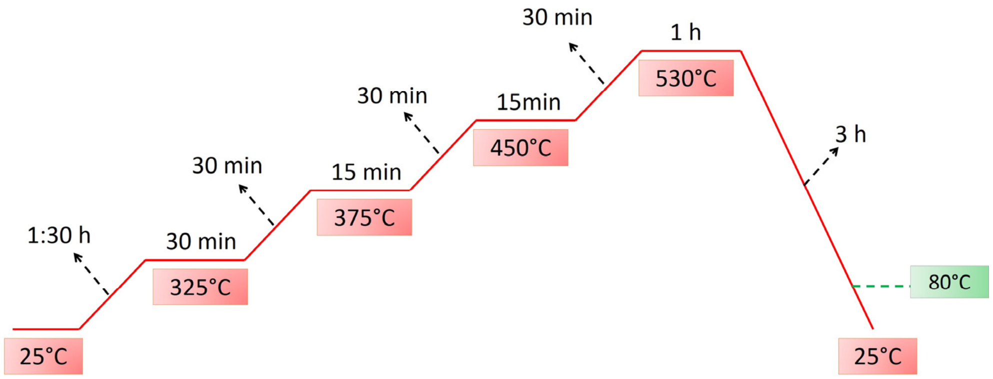

Figure S3. Temperature ramp for the sintering of TiO_2 films after screen printing deposition. When films are heat-treated prior to dye loading, the process is halted at 80 °C.

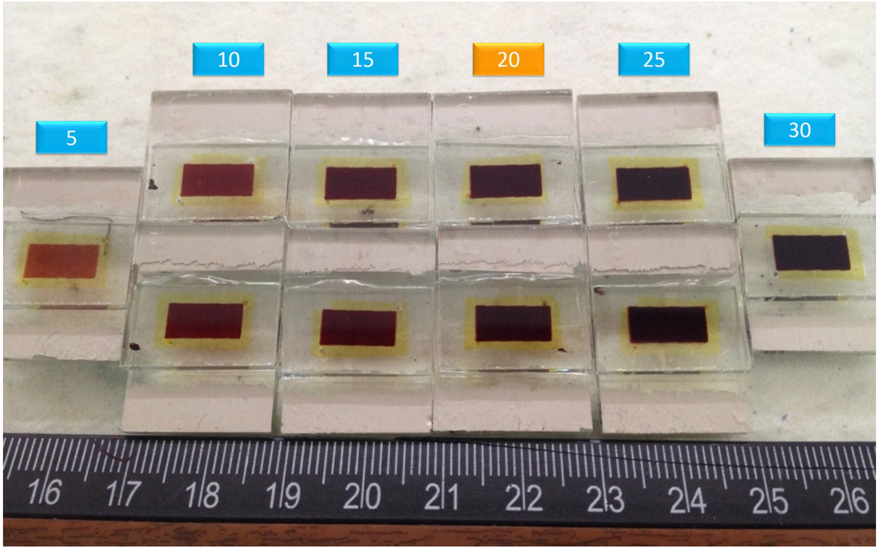

Figure S4. Dye solar cells prepared from the electrodes with a different number of screen-printed TiO_2 layers, hence, different thickness of the mesoporous, nanostructured TiO_2 film.

In Figure S5, R_S represents the series resistance of the FTO; R_{FTO} and C_{FTO} stand for the elements of resistance and capacitance for the recombination via the substrate uncovered by the TiO_2 nanoparticles; r_{tr} represents the resistance for electron transport along the metal oxide nanoparticles; r_r is the charge transfer resistance that exists to the process of recombination between electrons in the metal oxide and the redox species in the electrolyte solution (or dye); C_μ is the chemical capacitance; Z_d is the Warburg impedance of the diffusion of the redox species in the electrolytic solution; R_{pt} stands for the charge transfer resistance at the counter electrode/electrolyte interface and C_{pt} represents the Helmholtz capacitance at the counter electrode/electrolyte interface.

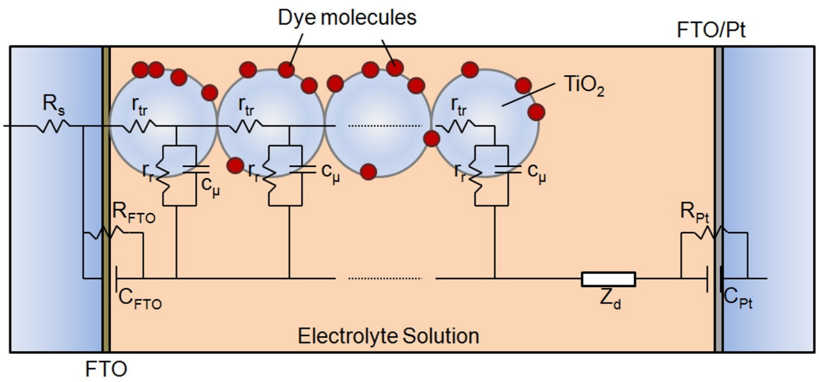

Figure S5. General equivalent circuit model used to fit impedance spectroscopy results for DSCs and to extract the electron transport and recombination parameters.

Figure S6 shows cross sections of the TiO_2 films, after sintering, as measured by profilometry. Different thicknesses were obtained, depending on the number of coatings and the characteristics of the paste. The thickest film obtained was around 32 μm.

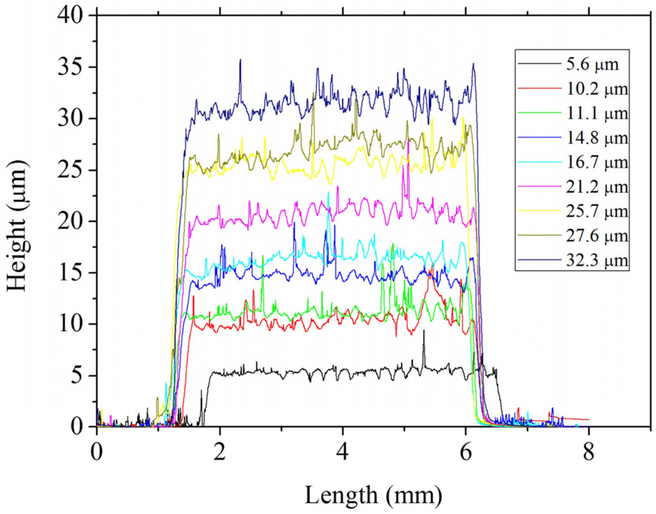

Figure S6. Cross sections of the TiO_2 films obtained by profilometry.

In Figure S7 SEM images of TiO_2 films of two different thickness are presented.

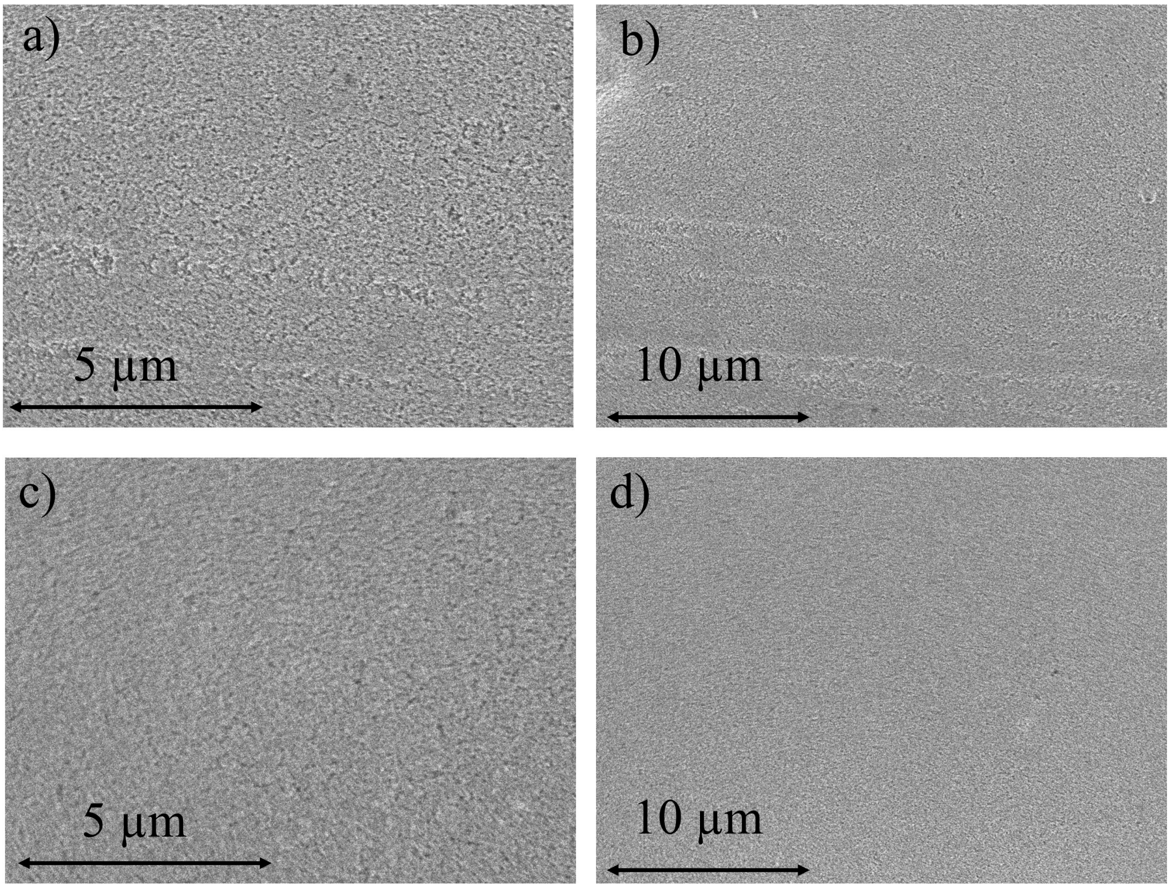

Figure S7. SEM images of TiO_2 films of different thicknesses: (**a**) 21.2 µm (magnification: 10,000×); (**b**) 21.2 µm (magnification: 5,000×); (**c**) 32.3 µm (magnification: 10,000×); and (**d**) 32.3 µm (magnification: 5,000×).

From Figure S7 we can observe that the deposition of the film was homogeneous and, although some imperfections can be observed, we obtained crack free films, even for film thicknesses larger than 30 µm.

Figure S8 shows the XRD patterns of the TiO_2 films, the red lines represent the main anatase phase peaks. It can be observed that anatase was the main phase present in the TiO_2 films, with a very small trace of brookite.

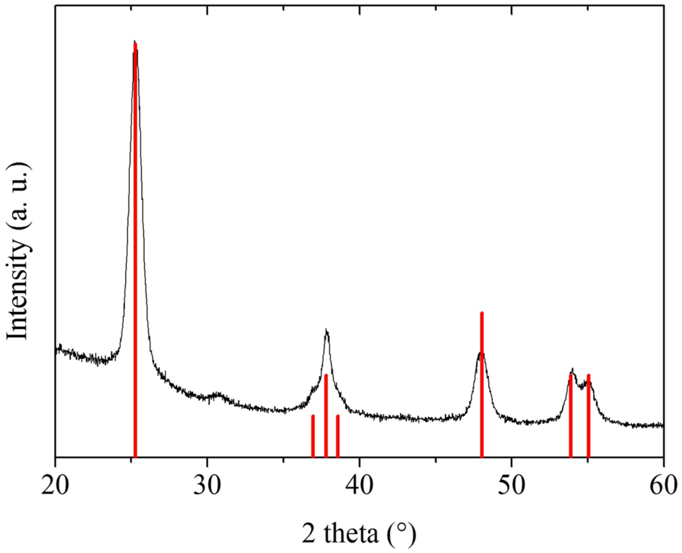

Figure S8. XRD pattern of the TiO_2 films. The main peaks of the anatase phase are shown.

The average crystallite size was estimated using the Scherrer equation [1]:

$$\tau = \frac{(0.94)\lambda}{B\cos\theta} \quad \text{(S2)}$$

where 0.94 is the Debye-Scherrer constant, λ is the X-ray wavelength, θ is the Bragg angle (measured in radians) at which the peak is observed, and B is the full width at half maximum. The average crystallite size calculated from both main peaks (2θ = 25.3° and 2θ = 48°) was approximately 18 nm.

Table S1. Parameters of the dye solar cells as a function of film thickness.

Thickness (µm)	J_{sc} (mA/cm^2)	V_{oc} (V)	*FF* (%)	Efficiency (%)
5.6	7.4	0.801	69	4.1
10.2	12.4	0.745	61	5.7
11.1	13	0.735	62	5.9
14.8	12.7	0.762	65	6.3
16.7	14.1	0.741	61	6.4
21.2	15	0.748	64	7.2
25.7	16	0.731	62	7.2
27.6	15.4	0.745	60	6.9
32.3	15.2	0.725	59	6.5

Figure S9 shows three different Nyquist diagrams for the same cell at three different voltages. The high frequency semicircle remains unchanged, which means no significant changes are occurring at the counter electrode/electrolyte solution interface. The middle frequencies semicircle, associated with transport and recombination of electrons in the mesoporous layer, increases as the voltage decreases. This is related to an increase in charge transfer resistance for lower voltages. This can be observed in the inset of Figure 3a). A third semicircle at low frequencies is observed for the high voltage solar cell. This implies diffusion limitation in this cell, due to the large thickness of the mesoporous layer. This semicircle disappears at lower voltages (*i.e.*, illumination intensity), which is usually the case for diffusion limitation systems.

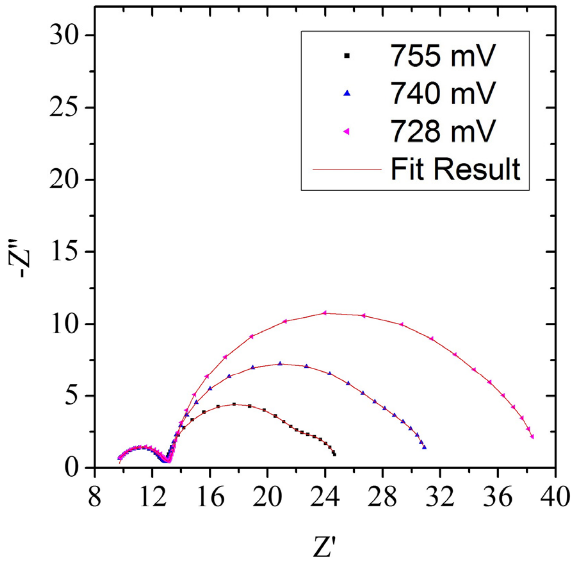

Figure S9. Nyquist plot and fitting results at three different voltages for the 21.2 µm cell.

Table S2. Parameters of the dye solar cells as a function of film thickness.

Label	J_{00} (A/cm³)	α (cm⁻¹)	m	Φ (cm⁻²·s⁻¹)	T	R_S (Ω)
A1	7.05×10^{-4}	3731.23	2	2.62×10^{17}	0.9	0
A2	7.05×10^{-5}	3731.23	2	2.62×10^{17}	0.9	0
A3	7.05×10^{-6}	3731.23	2	2.62×10^{17}	0.9	0
B1	7.05×10^{-5}	3731.23	1.5	2.62×10^{17}	0.9	0
B2	7.05×10^{-5}	3731.23	2	2.62×10^{17}	0.9	0
B3	7.05×10^{-5}	3731.23	2.5	2.62×10^{17}	0.9	0
C1	7.05×10^{-5}	3167.28	2	2.35×10^{17}	0.9	0
C2	7.05×10^{-5}	3731.23	2	2.62×10^{17}	0.9	0
C3	7.05×10^{-5}	1029.7	2	3.14×10^{17}	0.9	0

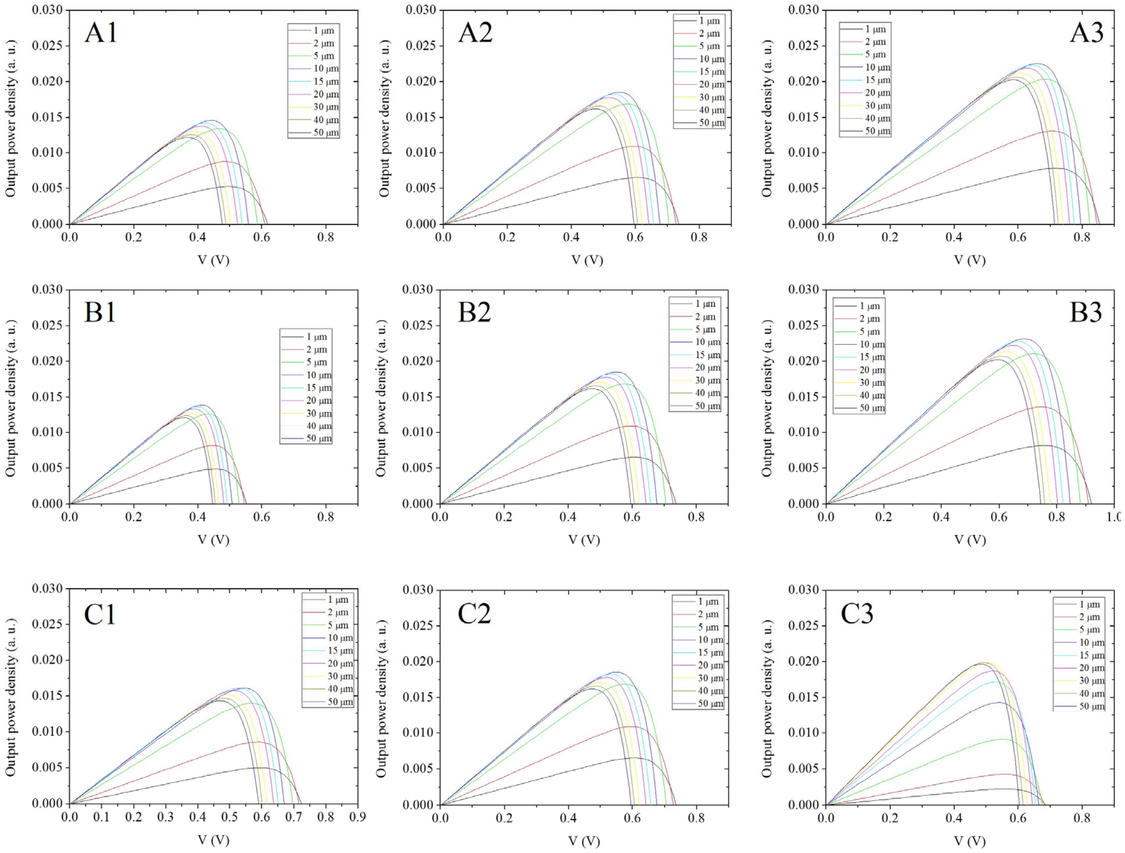

Figure S10. Power *versus* voltage plots for the A, B and C cells.

References

1. Anta, J.A.; Idigoras, J.; Guillen, E.; Villanueva-Cab, J.; Mandujano-Ramirez, H.J.; Oskam, G.; Pelleja, L.; Palomares, E. A continuity equation for the simulation of the current-voltage curve and the time-dependent properties of dye-sensitized solar cells. *Phys. Chem. Chem. Phys.* **2012**, *14*, 10285–10299.
2. Frank, J.A.; Kopidakis, N.; Lagemaat, J. Electrons in nanostructured TiO_2 solar cells: Transport, recombination and photovoltaic properties. *Coord. Chem. Rev.* **2004**, *248*, 1165–1179.
3. Barnes, P.R.F.; Anderson, A.Y.; Durrant, J.R.; O'Regan, B.C. Simulation and measurement of complete dye sensitised solar cells: Including the influence of trapping, electrolyte, oxidised dyes and light intensity on steady state and transient device behaviour. *Phys. Chem. Chem. Phys.* **2011**, *13*, 5798–5816.
4. Bisquert, J.; Fabregat-Santiago, F.; Mora-Seró, I.; Garcia-Belmonte, G.; Giménez, S. Electron lifetime in dye-sensitized solar cells: Theory and interpretation of measurements. *J. Phys. Chem. C* **2009**, *113*, 17278–17290.
5. Barnes, P.R.F.; Anderson, A.Y.; Juozapavicius, M.; Liu, L.; Li, X.; Palomares, E.; Forneli, A.; O'Regan, B.C. Factors controlling charge recombination under dark and light conditions in dye sensitised solar cells. *Phys. Chem. Chem. Phys.* **2011**, *13*, 3547–3558.

6. Zhu, K.; Kopidakis, N.; Neale, N.R.; van de Lagemaat, J.; Frank, A.J. Influence of surface area on charge transport and recombination in dye-sensitized TiO_2 solar cells. *J. Phys. Chem. B* **2006**, *110*, 25174–25180.
7. Shi, Y.; Dong, X. Coupled analysis of steady-state and dynamic characteristics of dye-sensitized solar cells for determination of conduction band movement and recombination parameters. *Phys. Chem. Chem. Phys.* **2013**, *15*, 299–306.
